# Genetic affinities of *Helicobacter pylori *isolates from ethnic Arabs in Kuwait

**DOI:** 10.1186/1757-4749-2-6

**Published:** 2010-07-05

**Authors:** M John Albert, Hanan M Al-Akbal, Rita Dhar, De Ronita, Asish K Mukhopadhyay

**Affiliations:** 1 Department of Microbiology, Faculty of Medicine, Kuwait University, Jabriya, Kuwait; 2 Microbiology Laboratory, Farwaniya Hospital, Farwaniya, Kuwait; 3 National Institute of Cholera and Enteric Diseases, Calcutta, India

## Abstract

*Helicobacter pylori *is one of the most genetically diverse of bacterial species, and since the 5'-end of *cagA *gene and the middle allele of *vacA *gene of *H. pylori *from different populations exhibit considerable polymorphisms, these sequence diversities were used to gain insights into the genetic affinities of this gastric pathogen from different populations. Because the genetic affinity of Arab strains from the Arabian Gulf is not known, we carried out genetic analysis based on sequence diversities of the *cagA *and the *vacA *genes of *H. pylori *from 9 ethnic Arabs in Kuwait. The analysis showed that the Kuwaiti isolates are closely related to the Indo-European group of strains, although some strains have a tendency to form a separate cluster close to the Indo- European group, but clearly distinct from East Asian strains. However, these results need to be confirmed by analyses of neutral markers (house-keeping genes in a multi-locus sequence typing [MLST]) platform. The profiling of virulence-associated genes may have resulted from ecologically distinct populations due to human migration and geographical separation over long periods of time.

## Findings

*Helicobacter pylori*, the Gram-negative, spiral, microaerobic bacterium, infects more than half of the world's population [1]. It is a major cause of gastritis and peptic ulcer disease, and a risk factor for gastric adenocarcinoma and mucosa-associated lymphoid tissue lymphoma [2]. There is extraordinary diversity among *H. pylori *strains as evident by DNA fingerprinting [3]. This diversity has been enhanced by extensive interstrain gene transfer and recombination [4,5]. In contrast, much stronger clonality, with the predominance of relatively fewer clones, is seen in populations of several other much-studied bacterial species [6,7]. The great diversity among *H. pylori *strains implies a striking lack of selection for just one or a few genotypes that might be best adapted for all humans.

The virulence associated *cagA*, and *vacA *genes provide examples in which evolutionary dynamics are likely to have been shaped by local differences in host physiology. CagA and VacA proteins each enter target cells and affect several normal cellular signal transduction pathways, with strengths and specificities that vary geographically.

The sequence diversities at the 5'-end of the *cagA *gene and the mid-region alleles of the *vacA *gene have been used to deduce the genetic relationships among strains from East Asia, South Asia, South America, Africa, Europe and USA [8,9]. However, there are no data on the genetic affinity of Arab strains from the Arabian Gulf Region including those from Kuwait, although some data exist on their genotypes [10,11]. In the current study, we analysed the genetic relationship of Kuwaiti strains with strains from other ethnic world populations. In order to investigate the gene pool diversity of Kuwaiti isolates of *H. pylori*, we sequenced putative virulence genes *cagA and vacA*, whose allele frequency have been shown to vary by ethnic group [12].

*H. pylori *isolates were cultured from the gastric biopsies of dyspeptic ethnic Arab Kuwaiti patients in a previous study on antibiotic susceptibility conducted during 2003-2005 [13]. The isolates were stored in brain heart infusion broth (Difco Laboratories, Detroit, Michigan, USA) with 15% glycerol at -70°C. The isolates were grown microaerobically on brain heart infusion agar supplemented with sheep blood, IsoVitalex and Dent supplement as described previously [13]. Chromosomal DNA was extracted by the CTAB (hexadecyl-trimethyl ammonium bromide) method [13] from culture derived from a single colony. A 219 bp fragment from the 5'-end of the *cagA *was amplified by PCR with the primers, cagA5 and cagA2 [9]. PCR was carried out in a 50-μl volume using 10 ng DNA, 25 μl of HotStarTaq Master Mix (catalogue number 203445, Qiagen, Valencia, California, USA), 10 pmol of each primer, for 40 cycles using the following conditions: 95°C for 60 s, 55°C for 60 s, and 72°C for 90 s. A 700 bp middle region of the *vacA *gene was amplified with the primers, VAm-F and VAm-R [9]. The concentrations of various PCR components were the same as in the *cagA *PCR, but amplification was done in 32 cycles with the following conditions: 94°C for 40 s, 55°C for 40 s, and 72°C for 70 s. The m1 (567 bp) or m2 (642 bp) allele of the middle region of the *vacA *gene was determined by PCR with the primers, VAG-F and VAG-R [14]. The concentration of PCR ingredients were the same as the other two PCRs above, but amplification was done in 35 cycles with the following conditions: 94°C for 60 s, 55°C for 60 s, and 72°C for 60 s. The PCR products were visualised by agarose gel electrophoresis after staining with ethidium bromide and examination under ultraviolet light. The PCR products were purified by High Pure PCR product purification kit (Roche Diagnostics, Indianapolis, Indiana, USA) according to manufacturer's instructions and directly sequenced. DNA sequencing was performed by the dideoxynucleotide chain termination method with an ABI PRISM BigDye Termination Cycle Sequencing Ready Reaction kit (Perkin-Elmer-Applied Biosystems, Forster City, California, USA) in an ABI PRISM automated sequencer. The DNA sequences were manually edited and compared with the selected sequences in the public database http://www.ncbi.nlm.nih.gov/nucleotide/ with the PHYLIP programme of J. Felsenstein http://evolution.genetics.washington.edu/phylip.html[15]. The genetic trees were generated using DDBJ programme http://www.ddbj.nig.ac.jp and visualised using Treeview programme (version1.61; http://taxonomy.zoology.gla.ac.uk/rod/treeview.html). The lengths of the *cagA *gene and the *vacA *gene used in the analyses were 209 bp and 620 bp (from the 700 bp amplified region) respectively.

In the previous study on the antibiotic susceptibility of *H. pylori *strains conducted in Al-Adan Hospital in Kuwait, cultures were available from 39 ethnic Arab Kuwaiti patients [13]. Of these cultures, 16 were amplified by the *cagA *gene primers, 21 by the *vacA *gene primers and 9 by primers for both the genes. Our study attempted to analyze the genomes of the 9 Kuwaiti isolates in defining the evolutionary history of these strains. The isolate numbers are: 7, 16, 21, 46, 202, 220, 43, 25, and 42675. The sequence obtained during this study have been assigned the following accession numbers [GenBank:GU212658 to GenBank:GU212666] (*cagA *5'-end), [GenBank:GU212667 to GenBank:GU212669 and GenBank:GU441847] (*vacA *m1 allele) and [GenBank:GU441844, GenBank:GU441846, GenBank:GU441848 to GenBank:GU441850] (*vacA *m2 allele).

**Figure 1:**
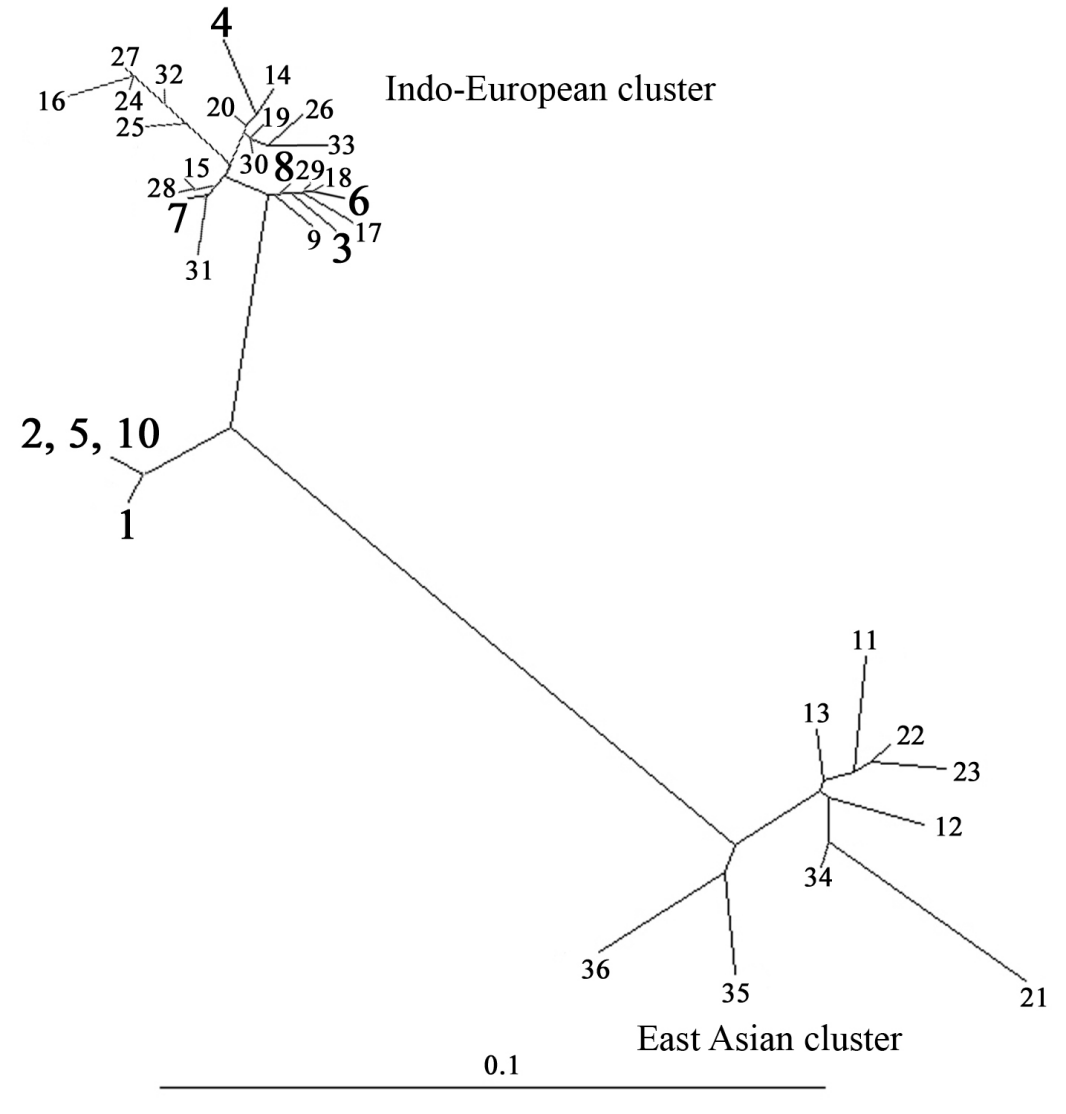
**Genetic tree based on an informative 209 bp segment at the 5' end of *cagA *of *H. pylori *strains determined in this study (test strains 1-8 and 10) or reported by others**. The test strains determined in this study are marked in black bold face. Sequences other than those for the test strains are from public database, as indicated. The tree was generated using DDBJ programme. Each number in this figure indicates the *cagA *gene sequence from a given strain, as follows (GenBank accession numbers in parentheses). The strains used are as follows. 1. 7 [GU212660], 2. 16 [GU212663], 3. 21 [GU212661], 4. 46 [GU212658], 5. 202 [GU212659], 6. 220 [GU212664], 7. 43 [GU212666], 8. 25 [GU212665], 9. 26695 (Britain) [NC_000915], 10. 42675 [GU212662], 11. ChinaR27 [AJ252979]; 12 ChinaR40 [AJ252982]; 13. ChinaR47 [AJ252985]; 14. Dutch79 [AJ252970]; 15. Dutch107 [AJ252963]; 16. Dutch161A [AJ252965]; 17. Dutch292 [AJ252971]; 18. Dutch419 [AJ252974]; 19. Gambia4659 [AF198468]; 20. Gambia 4797[AF198469]; 21. Hongkong77 [AF198485]; 22. Hongkong81 [AF198486]; 23. Hongkong97_42 [AJ239733];, 24. India3 [AF202219]; 25. India10 [AF202222]; 26. India18 [AF202224]; 27. India19[AF202225]; 28. Lithuania5_1 [AJ239734]; 29. Peru2B [AF198474]; 30. Peru4A [AF198477]; 31. Peru8C [AF198478]; 32. Peru24C [AF198473]; 33. Peru35B [AF198476]; 34. Thailand88-28 [AJ239722]; 35. JapanGC4 [AF198484]; 36. JapanF32 [AJ239726].

**Figure 2:**
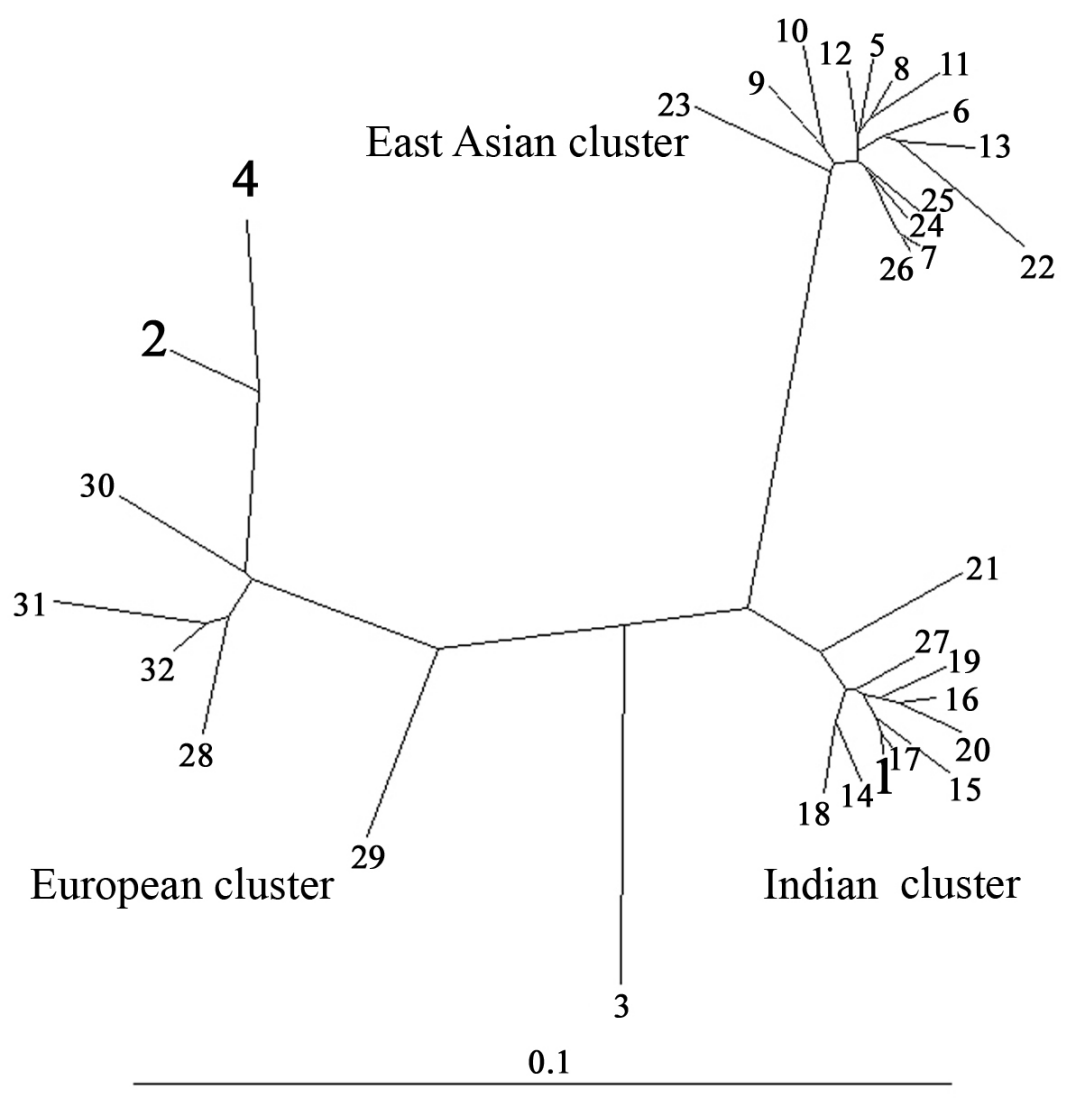
**Genetic tree based on informative 620 bp segment of *vacA *gene containing *vacAm1 *alleles of *H. pylori *strains determined in this study (strains 1-4) or reported by others**. The test strains determined in this study are marked in black bold face. Sequences other than those for the test strains are from public database, as indicated. The tree was generated using DDBJ program. Each number in this figure indicates the *vacAm1 *sequence from a given strain, as follows (GenBank accession numbers in parentheses). The strains used are as follows 1. 43 [GU212668], 2. 46 [GU212667], 3. 21 [GU441847] 4. 42675 [GU212669] 5. JapanF35 [AF049625]; 6. JapanF36 [AF049462]; 7. JapanF42 [AF049626]; 8. JapanF47 [AF049629]; 9. JapanF52 [AF049631]; 10. JapanF55 [AF049632]; 11. JapanF57 [AF049634]; 12. JapanF61 [AF049645] 13. JapanF63 [AF049635]; 14. JapanF64 [AF049647]; 15. India19 [AF220111]; 16. India48 [AF220112]; 17. India66 [AF220113]; 18. India89 [AF220114]; 19. India226 [AF220115]; 20. India227 [AF220116]; 21. India230 [AF220117]; 22. Mz19Germany [AJ006967]; 23. ChinaR13A [AF035610]; 24. ChinaR59A [AF035611]; 25. JapanF72 [AF049651]; 26. JapanF73 [AF049652]; 27. JapanF94 [AF049640]; 28. India18 [AF220110]; 29. NCTC11637 (Australia) [AF049653]; 30. NCTC11638 (Australia) [U07145]; 31. J99 (USA) [NC_000921]; 32. Poland 278 [AF097571].

**Figure 3:**
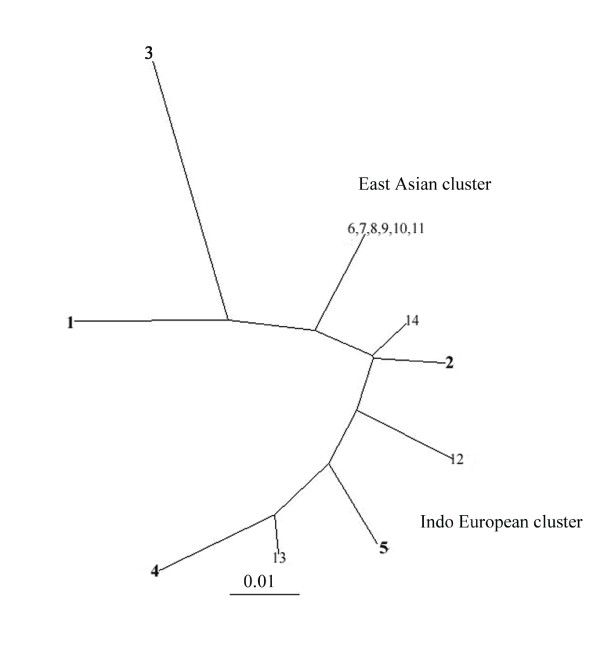
**Genetic tree based on an informative 620 bp segment of *vacA *gene containing *vacAm2 *alleles of *H. pylori *strains determined in this study (strains 1-5) or reported by others**. The test strains determined in this study are marked in black bold face. Sequences other than those for the test strains are from public database, as indicated. The tree was generated using DDBJ programme. Each number in this figure indicates the *vacAm2 *sequence from a given strain, as follows (GenBank accession numbers in parentheses). The strains used are as follows 1.7 [GU441846] 2.16 [GU441848] 3.25 [GU441849] 4.202 [GU441844] 5.220 [GU441850] 6. JapanOK160 [AB190979] 7. JapanOK204 [AB190986] 8. JapanOK205 [AB190987)] 9. JapanOK210 [AB190988] 10. JapanOK129 [AB190972] 11. JapanOK181 [AB190982] 12. GermanyMz28a [AJ006969] 13. GermanyMz26a [AJ006968] 14. India90 [AF220119].

To assess the genetic relationship between the strains isolated from Kuwait and those from other regions, we focused first on a segment near the 5' end of the *cagA *gene that had been used to distinguish East Asian and European strains [16]. This segment was amplified by PCR from nine Kuwaiti strains and sequenced directly. The sequences obtained were closely related to one another (97-99% match) and also to those from Western strains (91-99% DNA sequence match), essentially the Indo-European cluster, but only 77-86% related to those from Chinese and Japanese (East Asian) strains (Fig. 1). It was of interest to note that among the nine strains analysed from Kuwait, four of them (strains 7, 16, 202 and 42675) formed a somewhat separate cluster and were closely related to one another (99-100% match). These 4 strains were 92-93% related to the rest five Kuwaiti strains. Formal classification of these strains would require analysis from a larger number of strains from this region. DNA sequencing analysis of a 0.7-kb PCR fragment containing the *vacA *middle region from various parts of the world revealed the existence of 3 distinct groups (Fig. 2). The sequence analysis result showed that Kuwaiti isolates were 86-96% related to one another. Among the three isolates, one isolate (strain 43) was closely related to (96-99% match) Indian cluster, while the other two isolates (strain 46 and strain 4675) were 93-95% related to ethnic European strains and were 85-86% related to the East Asian cluster. This shows that strains isolated from Kuwait have an mixed gene pool. However, genetic analysis of the other five strains with *vacAm2 *genotype showed that they were much more scattered and related to the strains from Germany and India, even though the strain 25 was somewhat distant in contrast to the East Asian strains, which were much more congruent (Fig. 3).

*cagA *and *vacA *genes are virulence genes under selective pressure and hence subjected to sequence variation. Therefore, the data presented in this study need to be confirmed by sequence analyses of neutral genes such as house-keeping genes used in multi-locus sequence typing (MLST). Such analyses have been carried out for *H. pylori *isolates from various ethnic groups in India [17] and Iran [18]. These studies showed that isolates in these countries essentially belonged to the population of hpEurope, though isolates from various ethnic groups belonged to distinct subpopulation groups. Also, our conclusion of mixed gene in Kuwaiti isolates should be confirmed by Bayesian statistics.

The Arab population in the Middle East is a distinct ethnic race. Although the region is geographically separate, it established contacts with other regions of the world through colonisation and trade hundreds of years ago. Our study has shown that the strains that infect ethnic Arab Kuwaiti population is closely related to the Indo-European strains although some strains have a tendency to form a separate cluster close to the Indo- European group but far away from East Asian strains. This suggests a common ancestry of strains circulating in Kuwait, India and the European countries. The ancestry of these strains is different from that of the strains circulating in the Far East since they formed a distinct cluster. Human migration and geographical separation over long periods of time may have resulted in ecologically distinct populations of *H. pylori *infecting individuals in different continents. The profiling of virulence-associated genes like *cagA *and *vacA *provide examples in which evolutionary dynamics might have been shaped by local differences in environmental conditions and host physiology and may have resulted in ecologically distinct populations due to human migration and geographical separation over long periods of time. The remarkable differences found to-date between various Asian and European *H. pylori *populations in at least a few loci along with the present data from Kuwaiti strains should encourage further analyses of strains from relatively understudied geographic areas and human ethnic groups like Middle Eastern regions where human migration and geographical separation over long periods of time are prominent. Such "geographic genomics" may unearth new genes that affect human infection, increase our understanding of bacterium-host interactions in colonisation and disease, and impart new insights into the evolution of this diverse and globally distributed human pathogen.

## Competing interests

The authors declare that they have no competing interests.

## Authors' contributions

MJA and AKM conceived the study and drafted the manuscript. HMA, RD and RoD carried out the work. All authors read the manuscript and approved the final draft.
